# Cholesterol Selectively Regulates IL-5 Induced Mitogen Activated Protein Kinase Signaling in Human Eosinophils

**DOI:** 10.1371/journal.pone.0103122

**Published:** 2014-08-14

**Authors:** Mandy E. Burnham, Stephane Esnault, Elon C. Roti Roti, Mary E. Bates, Paul J. Bertics, Loren C. Denlinger

**Affiliations:** 1 Department of Biomolecular Chemistry, School of Medicine and Public Health, University of Wisconsin – Madison, Madison, WI, United States of America; 2 Department of Medicine, School of Medicine and Public Health, University of Wisconsin – Madison, Madison, WI, United States of America; UAE University, Faculty of Medicine & Health Sciences, United Arab Emirates

## Abstract

Eosinophils function contributes to human allergic and autoimmune diseases, many of which currently lack curative treatment. Development of more effective treatments for eosinophil-related diseases requires expanded understanding of eosinophil signaling and biology. Cell signaling requires integration of extracellular signals with intracellular responses, and is organized in part by cholesterol rich membrane microdomains (CRMMs), commonly referred to as lipid rafts. Formation of these organizational membrane domains is in turn dependent upon the amount of available cholesterol, which can fluctuate widely with a variety of disease states. We tested the hypothesis that manipulating membrane cholesterol content in primary human peripheral blood eosinophils (PBEos) would selectively alter signaling pathways that depend upon membrane-anchored signaling proteins localized within CRMMs (*e.g.*, mitogen activated protein kinase [MAPK] pathway), while not affecting pathways that signal through soluble proteins, like the Janus Kinase/Signal Transducer and Activator of Transcription [JAK/STAT] pathway. Cholesterol levels were increased or decreased utilizing cholesterol-chelating methyl-β-cyclodextrin (MβCD), which can either extract membrane cholesterol or add exogenous membrane cholesterol depending on whether MβCD is preloaded with cholesterol. Human PBEos were pretreated with MβCD (cholesterol removal) or MβCD+Cholesterol (MβCD+Chol; cholesterol delivery); subsequent IL-5-stimulated signaling and physiological endpoints were assessed. MβCD reduced membrane cholesterol in PBEos, and attenuated an IL-5-stimulated p38 and extracellular-regulated kinase 1/2 phosphorylation (p-p38, p-ERK1/2), and an IL-5-dependent increase in interleukin-1β (IL-1β) mRNA levels. In contrast, MβCD+Chol treatment elevated PBEos membrane cholesterol levels and basal p-p38, but did not alter IL-5-stimulated phosphorylation of ERK1/2, STAT5, or STAT3. Furthermore, MβCD+Chol pretreatment attenuated an IL-5-induced increase in cell survival at 48 hours, measured as total cellular metabolism. The reduction in cell survival following cholesterol addition despite unaltered STAT phosphorylation contradicts the current dogma in which JAK/STAT activation is sufficient to promote eosinophil survival, and suggests an additional, unidentified mechanism critically regulates IL-5-mediated human PBEos survival.

## Introduction

Human eosinophils are terminally differentiated granular leukocytes that contribute to inflammation and asthmatic airway remodeling, coronary artery disease, and Alzheimer's disease [1]–[10]. Eosinophils are activated as part of the immune system inflammatory response by cytokines released from mast and T helper 2 cells. The binding of extracellular interleukin (IL) 5 family cytokines (IL-5, IL-3, and Granulocyte Macrophage-Colony Stimulating Factor [GM-CSF]) activates inflammatory hallmarks in eosinophils, including prolonged survival, adherence, degranulation (release of cytotoxic proteins), and secretion of pro-inflammatory cytokines and chemokines [11]–[16]. In particular, IL-5 binding to its cognate receptor activates the mitogen activated protein kinase kinase/extracellular-regulated kinase (MEK/ERK), p38 mitogen activated protein kinase (MAPK), and Janus Kinase/Signal Transducer and Activator of Transcription (JAK/STAT) pathways [17]–[22] by inducing phosphorylation of the respective signaling proteins. Enhanced eosinophil survival promoted by these pathways is in turn characterized by up-regulation of pro-survival factors [23], [24], as well as down-regulation of pro-apoptotic factors like caspase 3 activation (cleavage) [25]–[27]. Cyclin family proteins, traditionally defined by their role in regulating cell division, interact with caspases [28] and may also influence survival, as cyclin D3 protein levels parallel eosinophil survival [19]. Eosinophil inflammatory response to stimuli requires proper synchronization of these numerous signaling events.

Eosinophil activation is ultimately the culmination of coordinating extracellular signals with downstream intracellular responses. One of the ways cells ensure specificity in coupling signal receipt with responses is by clustering signaling proteins based on cell membrane architecture, which is in turn determined by lipid distribution. Specificity can be achieved both by the inclusion and exclusion of signaling proteins to/from cholesterol-rich membrane microdomains (CRMMs), which are classically characterized by their resistance to detergents, and often referred to as lipid rafts. CRMMs cluster proteins that regulate signaling in most cells, including T cells, macrophages, and neutrophils [29]–[33], and specifically serve as docking and localization sites for membrane-anchored Src family kinases, which regulate MAPK signal-transduction [34]. These domains are fluid, allowing dynamic regulation, and their structure is dependent upon available cholesterol. As circulating cells, eosinophils are exposed to plasma cholesterol, and are likely to undergo cell membrane reorganization in response to disease states like hypercholesterolemia.

While studies have linked eosinophil-mediated inflammation with diseases caused or affected by abnormal cholesterol levels, including atherosclerosis and Alzheimer's, and recent studies have assessed the relationship between hypercholesterolemia and asthma [1], [5]–[8], [10], [35]–[41], little is known about how cholesterol directly regulates inflammatory signaling in eosinophils. The potential for CRMM disruption to provide separation of distinct intracellular responses to the same extracellular signal is highlighted by recent studies demonstrating that while the IL-5 receptors localize to both CRMMs and detergent-soluble membrane regions in eosinophils, the localization determines which intracellular signaling proteins are directly associated with the receptor [42], [43]. Lei and colleagues found that IL-5Rs localized to CRMMs co-immunoprecipitate with the membrane-anchored MAPK signaling cascade initiator, Lyn tyrosine kinase. These CRMM-localized IL-5Rs do not, however, co-immunoprecipitate with JAK2 [43], a soluble (cytosolic) tyrosine kinase initiator of the JAK/STAT pathway. Conversely, the IL-5Rs in the detergent-soluble portions of the membrane bind JAK2 but not Lyn [43]. These data predict that altering membrane composition by depleting or enhancing cholesterol levels would selectively disrupt pathways like MAPK which are dependent upon membrane-anchored signals, while leaving pathways like JAK/STAT, which utilize soluble proteins, intact following IL-5 binding to its cognate receptor. Manipulating cholesterol levels to selectively alter CRMM-dependent pathways downstream of IL-5 stimulation in primary human eosinophils may allow mechanistic separation of signaling pathways, mimic a variety of human disease states associated with both low and high serum cholesterol levels, and will enhance understanding of eosinophilic inflammation.

This study defines the consequences of membrane cholesterol depletion and addition on specific signaling proteins and inflammatory hallmarks in human PBEos. We tested the hypothesis that reducing cellular membrane cholesterol attenuates MAPK signaling and downstream physiological responses, but not JAK/STAT signaling. We found that reducing membrane cholesterol selectively down-regulated a subset of responses to IL-5, including reduced ERK1/2 and p38 phosphorylation, along with *IL-1β* mRNA induction. In contrast, depleting membrane cholesterol did not alter STAT3, STAT5, or cyclin D3 phosphorylation nor total cell metabolic activity following IL-5 stimulation. Furthermore, the addition of exogenous cholesterol basally activated p-p38 and led to reduced cellular metabolic activity 48 hours after IL-5 stimulation, despite maintained STAT phosphorylation. These data suggests an additional, unidentified cholesterol-dependent mechanism regulates IL-5-mediated human PBEos survival.

## Methods

### Human Eosinophil Isolation

Eosinophils were purified from peripheral human blood obtained from atopic patients under informed written consent. The University of Wisconsin-Madison Center for Health Sciences Human Subjects Committee approved this protocol.

Unless specified, all laboratory reagents were obtained from Sigma-Aldrich (St. Louis, MO, USA) or Fisher Scientific (Itasca, IL, USA). Heparinized blood was diluted with HBSS (Mediatech, Manassas, VA, USA) without Ca^2+^, and a granulocyte mixture was obtained from the leukocyte buffy coat after centrifugation, 20 min at 700× g, through a Percoll monolayer (1.090 g/ml) (Pharmacia, Piscataway, NJ, USA). Eosinophils were purified by negative selection as previously described [44]; recovered eosinophils were at least 97% pure and 98% viable as evaluated by Giemsa's-based Diff-Quik stain (Baxter Scientific Products, McGaw Park, IL, USA) and trypan blue exclusion, respectively.

### Cholesterol Treatments

**Figure 1:**
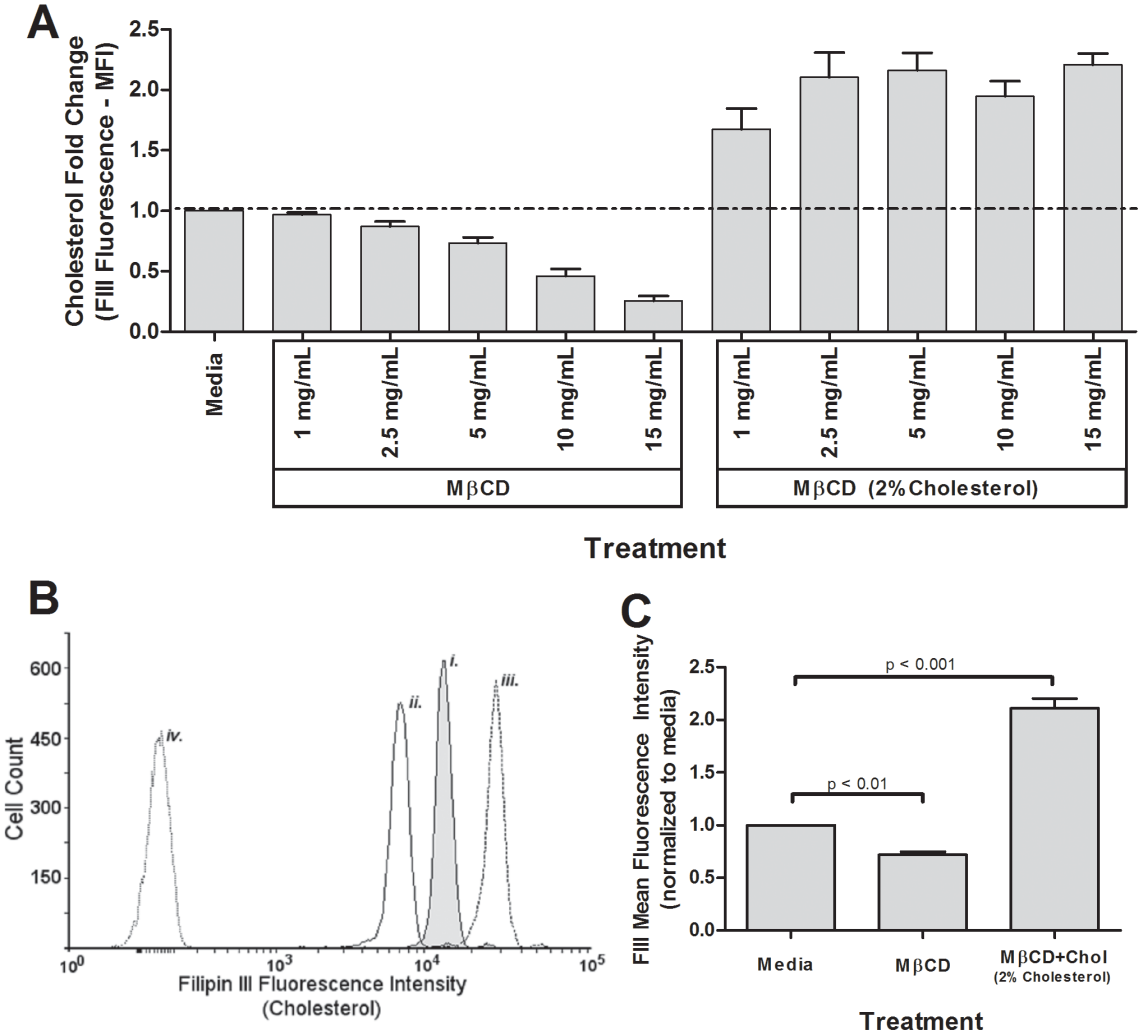
MβCD reduced cholesterol dose-wise in human peripheral blood eosinophils, while MβCD+Chol (2% cholesterol) elevated cholesterol. Three to five-hundred thousand eosinophils per treatment were treated 1 hour with media, MβCD, or MβCD+2%Chol (**A**–**C**) then fixed in 2% paraformaldehyde, stained with 50 µg/mL Filipin III (cholesterol stain), and analyzed via flow cytometry. (**A**) Pooled data from dose-wise treatment (n = 5). (**B**) Representative histogram from n = 16 experiments of cells stained with FIII after treated with *i*. media, *ii*. 5 mg/mL MβCD, *iii*. or 5 mg/mL MβCD+2%Chol, as well as unstained eosinophils (*iv.*). (**C**) Quantification of 16 independent experiments of FIII-stained eosinophils after treatment with 5 mg/mL MβCD or 5 mg/mL MβCD+2%Chol. Unmarked comparisons were non-significant.

Freshly isolated PBEos (10^6^ cells/100 µL) were pre-incubated in media (RPMI-1640 [ATCC; Manassas, VA, USA] supplemented with 25 mM HEPES and 0.1% human serum albumin [Irvine Scientific; Santa Ana, CA, USA]) for 30 min followed by a 1-hour treatment with media, the cholesterol chelator MβCD (5 mg/mL), or soluble cholesterol (MβCD+Chol, 5 mg/mL), which is MβCD pre-loaded with cholesterol (or concentrations indicated in Figure 1 legend). All incubations were at 37°C.

### Eicosapentaenoic Acid Treatment

EPA (Sigma E7006, *cis*-5,8,11,14,17-Eicosapentaenoic acid) was supplied as a liquid, and diluted to final concentration in culture media. Freshly isolated PBEos were cultured in (RPMI-1640, 10 mM HEPES, 2 mM L-glutamine, 1% penicillin/streptomycin, 1% ciprofloxacin, 10% FBS) for 2 hours prior to the addition of 33 uM EPA or control-media, and overnight culture (18 hours).

### Flow Cytometry

To measure cell membrane cholesterol, human PBEos treated +/− MβCD or MβCD+Chol were fixed with 2% paraformaldehyde, then, stained with 50 µg/mL filipin III (FIII) for 1 hour. To compare receptor expression, MβCD or MβCD+Chol treated PBEos were immediately stained with 10 µg/mL PE-conjugated anti-IL-5Rα (BD Biosciences, Franklin Lakes, NJ, USA) or 2.5 µg/mL PE-conjugated anti-IL-5Rβ (eBiosciences, San Diego, CA, USA), then fixed with 2% paraformaldehyde in media. Cells were suspended in 1× PBS (pH 7.4) supplemented with 0.25% BSA, 1 mM Na_2_EDTA. Ten thousand events within the PBEos-defined forward and side-scatter gate were collected using a BD Biosciences (San Jose, CA, USA) LSRII flow cytometer (355 nm laser [525/50 filter] for FIII, and 561 nm laser [582/15 filter] for PE). Data analyses were performed using CellQuest software (BD Biosciences; Franklin Lakes, NJ, USA) and Flowing Software 2 (Turku Centre for Biotechnology, University of Turku, Finland). Flow data are summarized as the median fluorescence intensity (MFI).

### Immunoblotting

Human PBEos were pretreated for 1 hour with media +/− indicated concentrations of MβCD, MβCD+Chol, or EPA, then stimulated with media +/− 1 nM IL-5 (R&D Systems; Minneapolis, MN, USA) for indicated times at 37°C. Samples were diluted in ice-cold buffer A (20 mM Tris, pH 7.4, 137 mM NaCl, 1 mM EDTA, 1 mM Na_3_VO_4_, 20 mM β-glycerophosphate, 10 mM NaF, and [mammalian] protease inhibitor cocktail from Sigma), pelleted by centrifugation at 4°C, and lysed via vortexing in buffer A plus detergents (1% Triton X-100, 0.25% deoxycholate, and 0.1% SDS) at a concentration of 4×10^7^ cells/mL.

For cyclin D3 and caspase 3, treated and stimulated PBEos were placed on ice, pelleted by centrifugation, and lysed in buffer B (1% NaDodSO_4_ [SDS]), 10 mM DTT, 0.5 mM Na_3_VO_4_, 1 mM EDTA, 10% glycerol, 10 mM Tris, pH 8.0) at a concentration of 4×10^7^ cells/mL. Lysates were boiled for 5 minutes, sonicated 4 times, and held at −80°C for 15 minutes.

Following either lysis protocol, Western blotting was performed as previously reported [44]. Briefly, lysate insoluble material was removed via centrifugation, and lysates were loaded at 0.6–1×10^6^ cells per well on 10%, 12.4%, or 15% SDS-PAGE gels as appropriate. Blots were incubated according to manufacturer instructions with appropriate antibodies: phospho-specific ERK1/2 [pY185/187], phospho-specific p38 [pTpY180/182] (Invitrogen; Eugene, OR, USA), phospho-specific STAT5 [pY694], phospho-specific STAT3 [pY705] (Cell Signaling; Billerica, MA), anti-C4/Actin (BD Biosciences; Franklin Lakes, NJ, USA), anti-cyclin D3 (Santa Cruz Biotechnology, Santa Cruz, CA, USA), or anti-caspase 3 (Cell Signaling; Billerica, MA, USA) followed by appropriate HRP-conjugated secondary antibodies (Santa Cruz Biotechnology; Santa Cruz, CA, USA). Band quantification was performed using ImageJ software (National Institutes of Health, USA). Due to tight binding of phospho-specific antibodies and subsequent removal of protein upon stripping, actin was used as a loading control for all Western blots.

### Cell Viability and Metabolic Activity

Short-term cellular viability was measured via trypan blue exclusion. Briefly, following 4 or 24 hour continuous treatment with MβCD or MβCD+Chol, a cell suspension was mixed 1∶1 with trypan blue and mixed gently via pipetting 20 times. Live cells (those excluding trypan blue) were counted using a hemocytometer and used to calculate percent viable compared to total cell count.

To assess metabolic activity, human PBEos pretreated 1 hour with media, MβCD, or MβCD+Chol, washed, and cultured in triplicated +/−100 pM IL-5 for 48 hours. Afterwards, Phenazine methosulfate (PMS) and MTS (3-[4,5-dimethylthiazol-2-yl]-5-[3-carboxymethoxyphenyl]-2-[4-sulfophenyl]-2H-tetrazolium [Promega; Madison, WI, USA]) were added (final concentrations 7.3 µg/mL and 0.23 mg/mL, respectively). The metabolism of MTS to formazan was quantified by measuring the OD at 490 nm after 1-hour incubation.

### Isolation of RNA and subsequent qPCR

Purified PBEos were cultured 1 hour +/− MβCD or MβCD+Chol, then, stimulated with media +/−1 nM IL-5 for 4 hours. Total RNA was extracted (RNeasy Mini Kit [Qiagen; Valencia, CA, USA]), and the reverse transcription reaction was performed using the Superscript III system (Invitrogen/Life Technologies; Grand Island, NY, USA). mRNA expression was determined by qPCR using SYBR Green Master Mix (SABiosciences; Frederick, MD, USA). Human *IL-1β* specific primers (forward primer: TCGAGGCACAAGGCACAACAGG; reverse primer: CCATGGCTGCTTCAGACACTTGAGC) were used to quantify *IL-1β* mRNA levels, using the reference gene, β-glucuronidase to normalize. Data are expressed as fold change using the comparative cycle threshold (ΔΔCT) method as described previously [45], and values given are fold change (2-ΔΔCt) compared to the level in media-pretreated, non-stimulated eosinophils, which was set at 1 (n = 5).

### Statistical Analysis

Mean fluorescence intensity, protein band densitometry, and OD measurements were compared among treatment groups (media, MβCD, MβCD+Chol) and stimulants (media +/− IL-5). Where appropriate, measures were log or square root-transformed to achieve normal distributions and homogeneity of variance for ANOVA. A two-sided p-value<0.05 was regarded as statistically significant.

## Results

### MβCD and MβCD+Chol alter eosinophil membrane cholesterol levels in a dose-dependent manner

To test the hypothesis that the level of membrane-bound cholesterol alters signaling in primary human PBEos, we manipulated cholesterol content *in vitro* utilizing MβCD and MβCD+2% Cholesterol (w/w) (MβCD+2%Chol). Empty MβCD is a cholesterol chelator and depletes membrane cholesterol from the cells, whereas MβCD preloaded with cholesterol can deposit exogenous cholesterol into the cell membrane. Filipin III (FIII) is a fluorescent polyene antibiotic that stoichiometrically binds membrane-integrated cholesterol in a 1∶1 ratio [46], and is detectable via flow cytometry. Therefore, FIII serves as powerful tool for rapidly comparing relative membrane cholesterol levels in cell populations [47]–[50]. To define dose-dependent changes in cellular membrane cholesterol, FIII fluorescence was measured in purified human PBEos treated with step-wise doses of MβCD or MβCD+2%Chol. MβCD-treated human PBEos displayed dose-dependent decreases of FIII fluorescence relative to media-controls, reflecting a loss of membrane cholesterol. Treating PBEos with increasing concentrations of MβCD+2%Chol saturated cellular membrane cholesterol levels, reflected by a plateau in FIII fluorescence (Figure 1A). FIII concentration was not a limiting factor since doubling the FIII concentration did not further increase the FIII fluorescent signal after any of the treatment conditions (data not shown). Relative to media control, treatment with 5 mg/mL MβCD significantly decreased FIII median fluorescent intensity (MFI) by ∼25 (p<0.01, n = 15; Figure 1B and C,). Conversely, 5 mg/mL MβCD+2%Chol treatment significantly increased FIII MFI ∼2× (Figure 1B, C) (p<0.001, n = 15). These data indicate MβCD treatment quantitatively alters membrane cholesterol level in PBEos. Neither 5 mg/mL MβCD nor MβCD+2%Chol treatment altered PBEos size or density as indicated by forward and side scatter, respectively (Figure S1). Based on trypan blue viability measurements, neither 5 mg/mL MβCD or MβCD+2%Chol significantly altered cell viability 4 and 24 hours post-treatment (data not shown), and these doses were therefore selected as general working concentrations for the remainder of the study.

### IL-5 receptor subunit surface expression was independent of membrane cholesterol content

**Figure 2:**
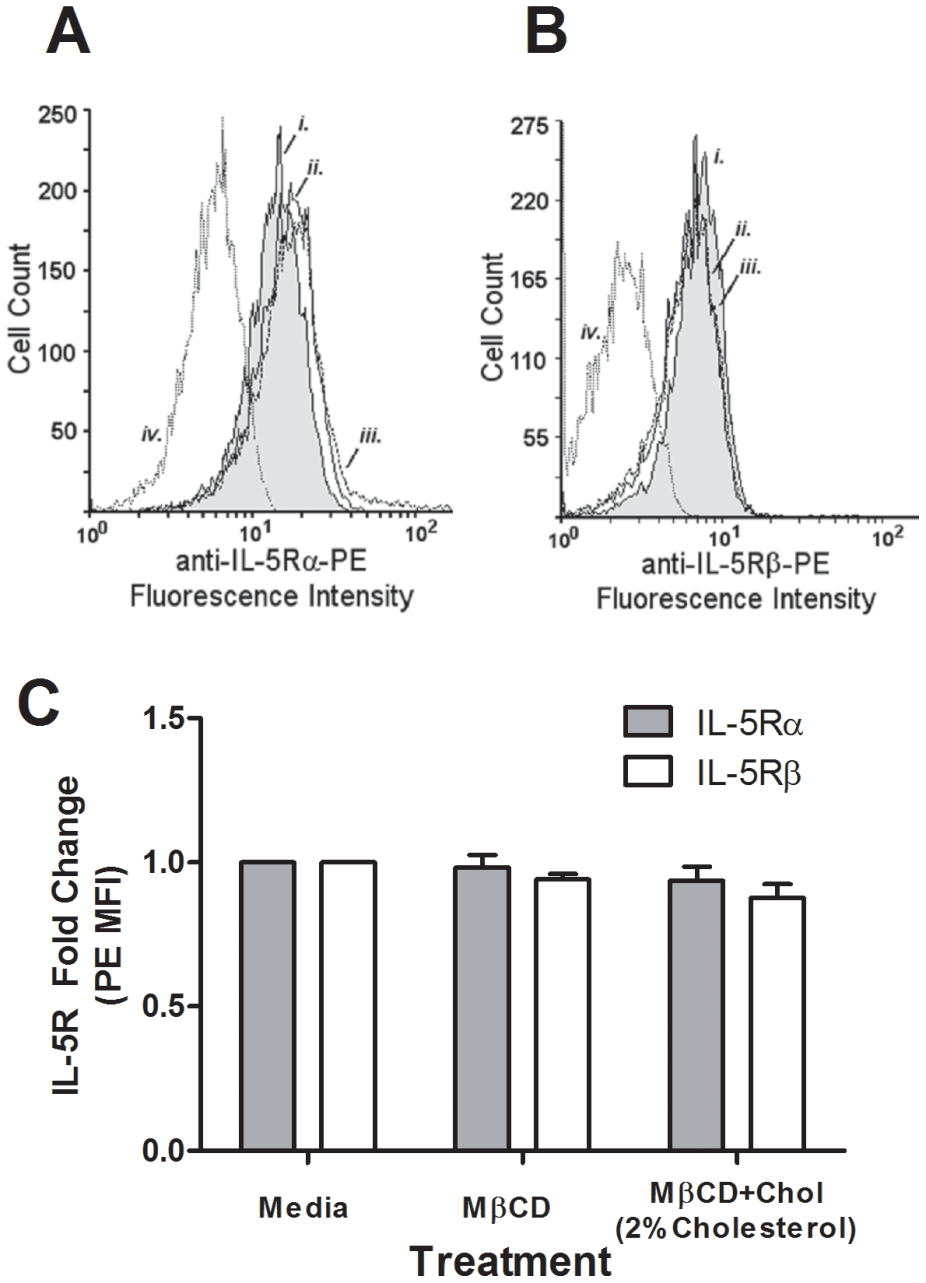
Membrane cholesterol manipulation did not alter IL-5Rα or β surface expression. Representative histograms of PBEos treated 1 hour with media, MβCD, or MβCD+2%Chol (3–5×10^5^ per treatment) then stained with (**A**) PE-conjugated anti-IL-5Rα (n = 9) or (**B**) PE-conjugated anti-IL-5Rβ (n = 3). (**C**) Pooled data from receptor staining: grey bars, IL-5Rα (n = 9), open bars, IL-5Rβ (n = 3). Error bars indicate SEM, p-values from one-way ANOVA. Unmarked comparisons were non-significant.

Altering receptor surface expression levels is one way in which cells dynamically alter sensitivity to extracellular ligands, and hence subsequent intracellular signaling. To determine whether altered membrane cholesterol levels influence PBEos surface expression of the IL-5-specific receptor subunit α (IL-5Rα) and the IL-5 family common β chain subunit (IL-5Rβ), which dimerize to form the functional IL-5 receptor, respective surface expression levels were measured via flow cytometry in unpermeabilized cells following treatment with MβCD or MβCD+2%Chol (Figure 2A and B respectively). No significant changes in MFI were observed across treatments for IL-5Rα (Figure 1C, n = 9, p>0.05) or IL-5Rβ (Figure 1C, n = 3, p>0.05). These data indicate that neither MβCD nor MβCD+2%Chol treatment altered IL-5Rα or IL-5Rβ surface expression.

### IL-5 induced STAT phosphorylation was independent of cellular membrane cholesterol

**Figure 3:**
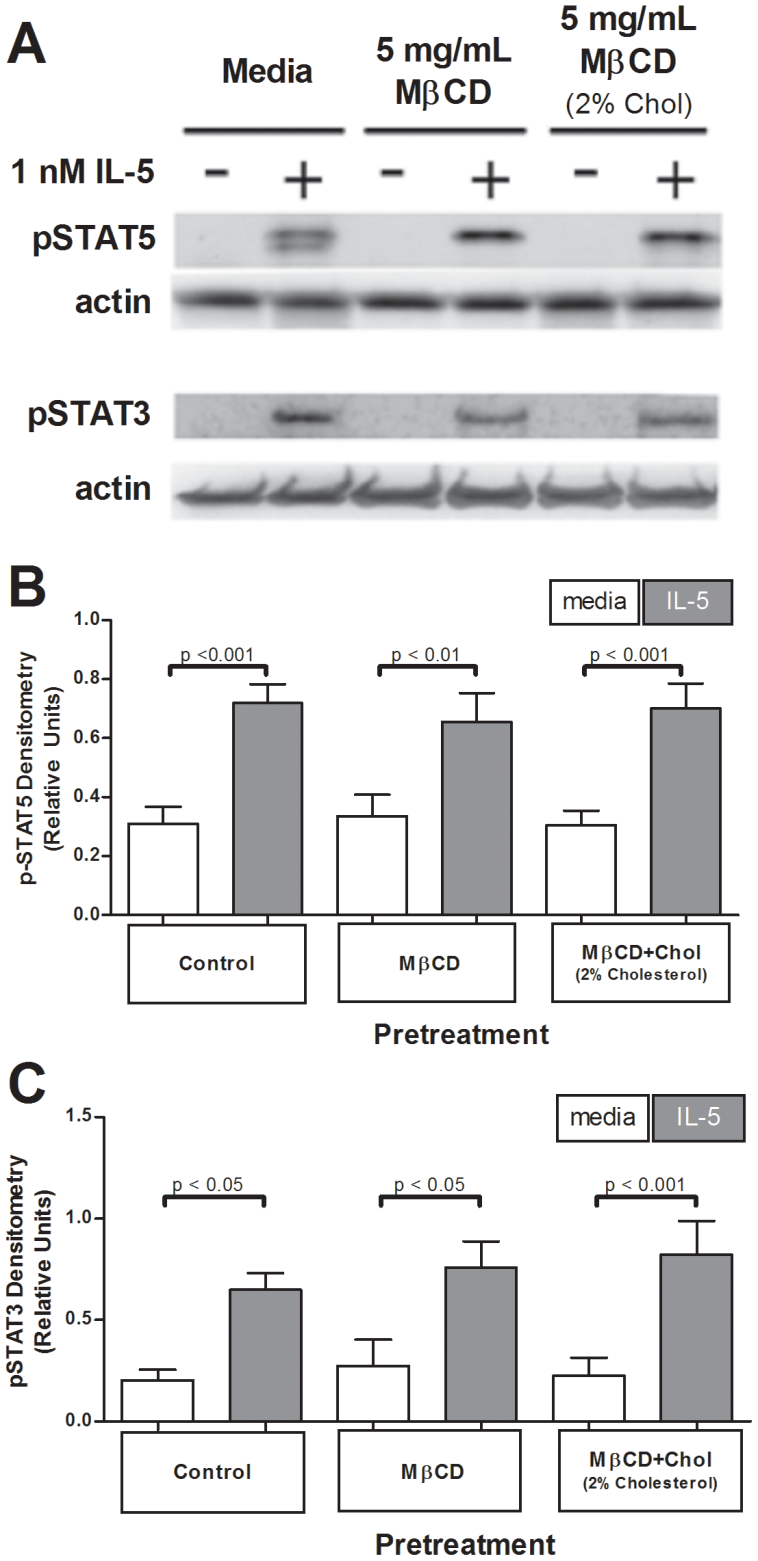
Membrane cholesterol manipulation did not affect IL-5-induced p-STAT5 or p-STAT3. One million eosinophils per treatment pretreated 1 hour with media, 5 mg/mL MβCD, or 5 mg/mL MβCD+2%Chol, were stimulated +/− IL-5 for 15 min. (**A**) Samples were immunoblotted for active STAT5 or STAT3. (**B**) Data were pooled from 19 experiments (p-STAT5) or (**C**) 5 experiments (p-STAT3) and normalized to actin loading; pooled data were square root transformation for normalcy. Error bars indicate SEM, p-values from one-way ANOVA. Unmarked comparisons were non-significant.

Given unchanged IL-5Rα and IL-5Rβ surface expression in human PBEos following cholesterol manipulation, alterations in downstream IL-5-induced signaling would likely be due to the effects cellular cholesterol levels have on proteins that transduce signal from the receptor through intracellular pathways rather than total receptor expression. We therefore assessed cholesterol-mediated changes in activation of the JAK/STAT, MEK/ERK, and p38-MAPK pathways by quantifying the phosphorylated (activated) state of representative signaling proteins from each pathway after cholesterol deposition or depletion. Unlike MAPK pathways, JAK/STAT signal transduction is mediated by soluble proteins, and functions independently of membrane-anchored signaling proteins localized to CRMMs. We therefore predicted that cholesterol modulation would not alter JAK/STAT signaling. As indicated in Figure 3A–C, neither MβCD (cholesterol depletion) nor MβCD+2%Chol (cholesterol addition) altered IL-5-stimulated phosphorylation of STAT5 (p-STAT5, n = 15) or STAT3 (p-STAT3, n = 5). These data indicate that signaling through the JAK/STAT pathway in PBEos was independent of membrane cholesterol levels.

### IL-5-induced ERK and p38 phosphorylation are selectively disrupted by altered membrane cholesterol levels in human eosinophils

**Figure 4:**
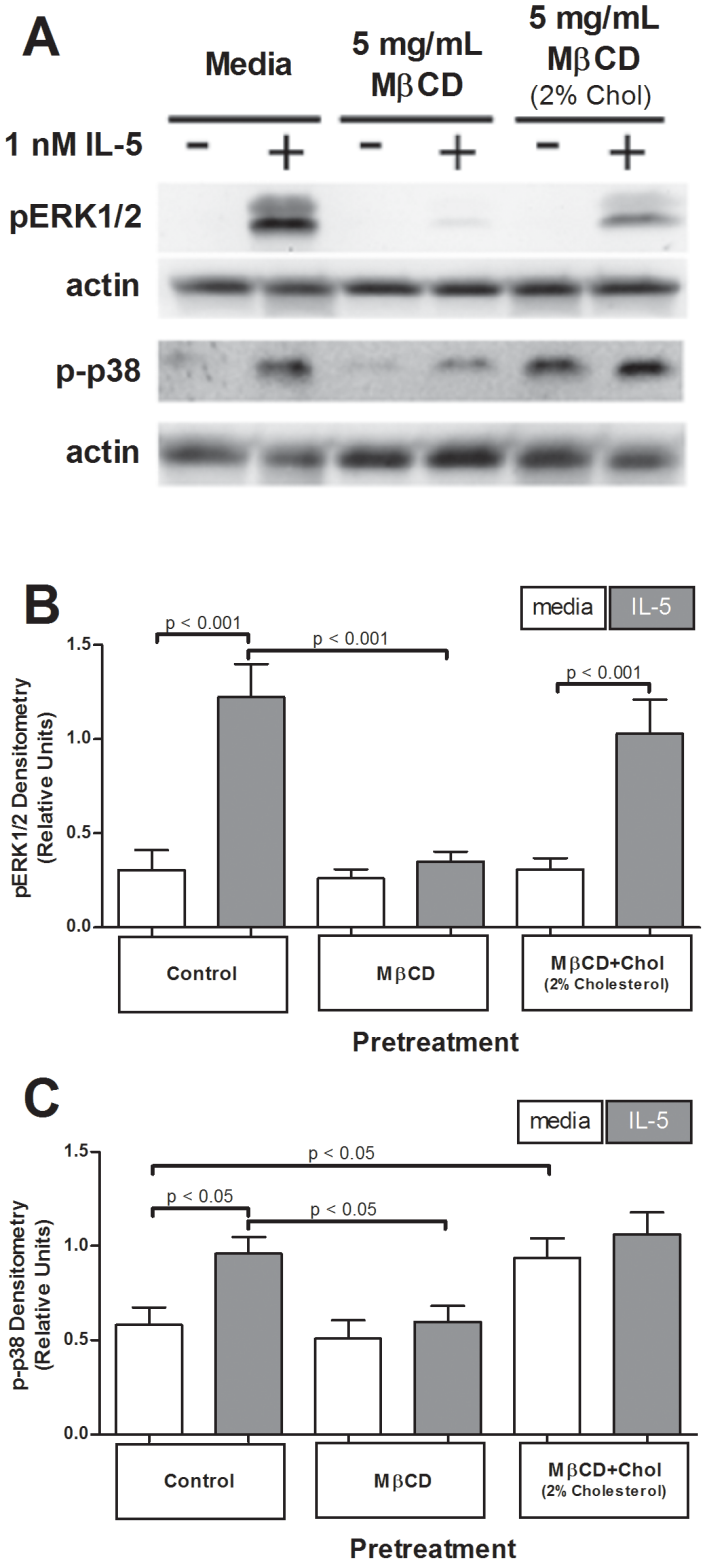
Cholesterol depletion attenuated IL-5-induced phosphorylated ERK1/2 and p38, while cholesterol addition enhanced basal p38 phosphorylation. One million eosinophils per treatment pretreated 1 hour with media, 5 mg/mL MβCD, or 5 mg/mL MβCD+2%Chol, were stimulated +/− IL-5 for 15 min. (**A**) Samples were immunoblotted for active ERK1/2 or p38. (**B**) 14 experiments (ERK1/2) or (**C**) 16 experiments (p38) normalized to actin loading; pooled data were square root transformation for normalcy. Error bars indicate SEM, p-values from one-way ANOVA. Unmarked comparisons were non-significant.

Our model predicted cholesterol depletion would attenuate IL-5-induced MAPK signaling (*e.g.*, MEK/ERK and p38), because both pathways are activated by membrane-anchored kinases. Human PBEos pretreated with media, MβCD, or MβCD+2%Chol were stimulated with IL-5 for 15 min, and lysates were immunoblotted for phosphorylated ERK1/2 and p38. Figure 4A and B demonstrate MβCD pretreatment attenuated IL-5-induced ERK1/2 phosphorylation compared to media-pretreated, IL-5-stimulated controls (p<0.001, n = 13; Figure 4B). MβCD pretreatment similarly attenuated an IL-5-induced increase in p-p38 levels (p<0.05, n = 16; Figure 4A and C). Cholesterol treatments did not alter total ERK1/2 or p38 protein expression levels (data not shown). These data demonstrate that cholesterol depletion decreased IL-5-induced activation of MEK/ERK and p38 pathways.

To determine whether adding exogenous cholesterol, predicted to enhance CRMM formation, would have the opposite effect and increase ERK/p38 phosphorylation, PBEos were treated with MβCD+2%Chol and lysates assessed for p-ERK/p38. Pretreatment with MβCD+2%Chol did not significantly change total levels of IL-5-induced p-ERK1/2 or p-p38 compared to media-treated, IL-5 stimulated controls (p>0.05, n = 13, n = 16, respectively; Figure 4A–C). Interestingly, the addition of exogenous cholesterol increased basal p38 phosphorylation levels relative to media-pretreated, media-stimulated control eosinophils (p<0.05, n = 16; Figure 4A and C). Furthermore, while IL-5 continued to potentiate p-ERK1/2 relative media-control after MβCD+2%Chol (3× increase, p<0.001, n = 13, Figure 4A and B), cholesterol addition limited the dynamic range of the p-p38 response. The increase in basal p38 phosphorylation eliminated the IL-5-induced increase in p-p38 in MβCD+2%Chol-treated cells, (p>0.05, n = 16; Figure 4A and C). Therefore the fact that PBEos receiving exogenous cholesterol exhibited the same levels of p38 phosphorylation as control cells post-IL-5 was due to elevated basal p38 phosphorylation, rather than IL-5 stimulation. These data indicate that increasing membrane cholesterol levels did not alter overall phosphorylation of MEK/ERK pathway proteins following IL-5 stimulation, but increased basal p38 phosphorylation and eliminated the p38 response to IL-5.

To control for effects of exposing cells to MβCD, PBEos were treated with MβCD+1% Cholesterol (MβCD+1%Chol), which resulted in a no-net change in eosinophil membrane cholesterol levels as quantified via FIII signal in flow cytometry (n = 5; Figure S2A and B). PBEos pretreated with MβCD+1%Chol exhibited no differences in basal or IL-5-stimulated p-p38 levels compared with media pretreated, IL-5-stimulated controls (n = 5; Figure S2C and D). MβCD+1%Chol treatment similarly did not alter total STAT3 or STAT5 protein expression levels (data not shown).

While p38 phosphorylation was clearly regulated by membrane cholesterol, MβCD treatment alone cannot distinguish whether the phosphorylation events are specifically dependent upon lipid raft organization, or total cholesterol content. To begin to separate these mechanisms, we treated cells with eicosapentaenoic acid (EPA), a polyunsaturated fatty acid which can redistribute cholesterol from lipid rafts across the membrane, and disrupts nascent lipid raft architecture [51]–[54]. If the regulation of p38 phosphorylation was solely dependent upon total membrane cholesterol, EPA treatment was predicted to have no effect. Changes in p38 phosphorylation following EPA treatment, however, would indicate the signaling event is dependent upon lipid raft organization. PBEos were cultured overnight (18 hours) in the presence or absence of 33 uM EPA, followed by 15 min. stimulation with 1 nM IL-5. Western blots of lysates probed with p-p38 antibodies revealed that EPA treatment increased basal p38 phosphorylation in a manner parallel to MβCD+2%Chol (Fig. 4), such that there was no IL-5 induced increase, and total p-p38 levels were similar to media/IL-5 treated cells (Fig. S3A, B, n = 3). As a control, lysates were also probed for p-STAT5; EPA did not change IL-5-induced increases in p-STAT5 (Fig. S3A, C, n = 3), consistent with the fact this pathway is regulated by soluble proteins. Baseline levels of p-STAT5 were undetectable in these experiments, and therefore not included in quantification (Figs. S3A, C). These data demonstrate that p38 phosphorylation was dependent upon lipid raft architecture of the plasma membrane, independently of total cholesterol content.

### Reduced cellular membrane cholesterol attenuated IL-5-induced increase in IL-1β mRNA

**Figure 5:**
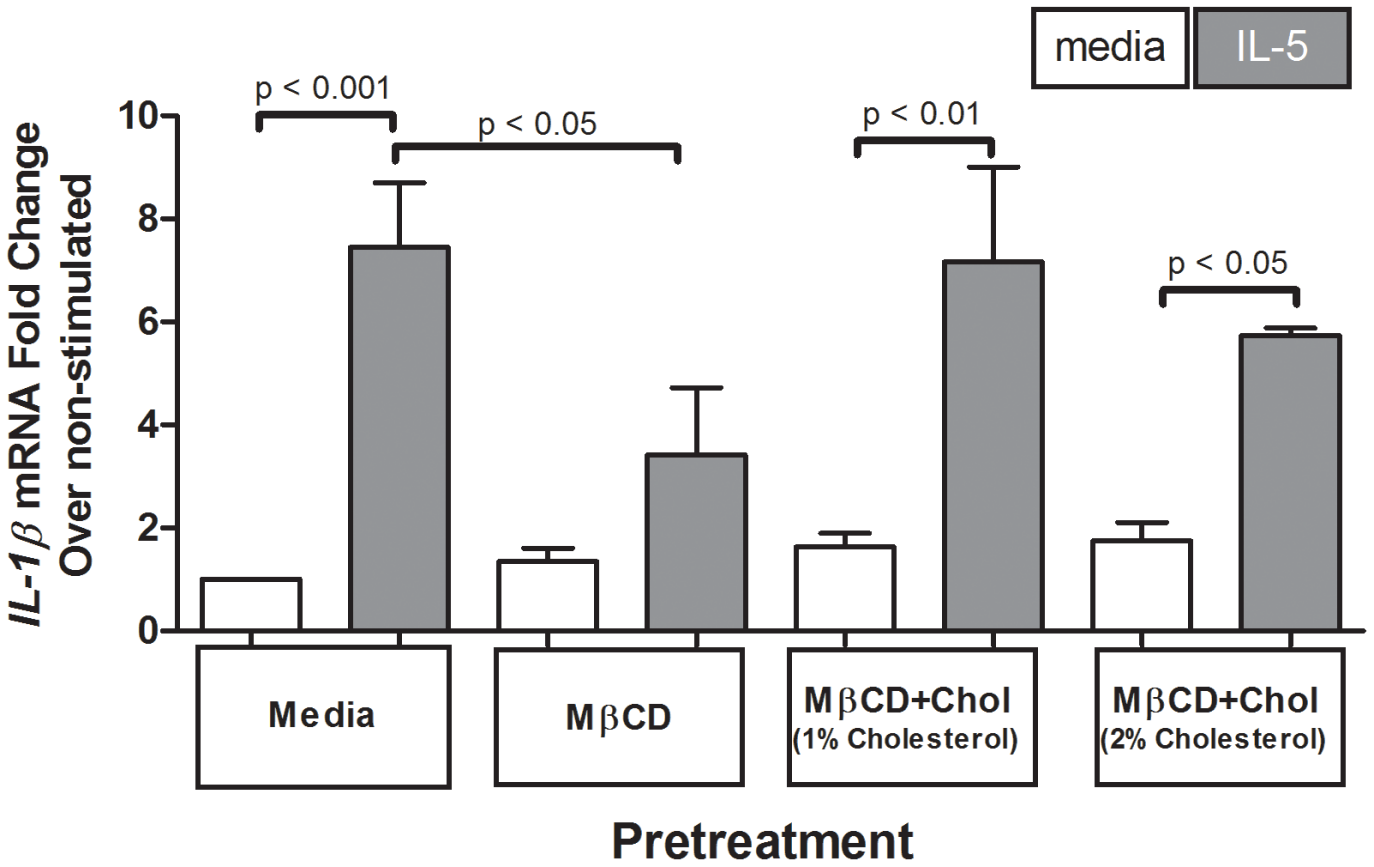
Cholesterol depletion decreased IL-5-induced *IL-1β* mRNA. One million eosinophils per treatment were pretreated 1 hour with media, 5 mg/mL MβCD, 5 mg/mL MβCD+1%Chol, or 5 mg/mL MβCD+2%Chol, then stimulated +/−1 nM IL-5 for 4 hours. Sample lysates were assessed for *IL-1β* mRNA. Pooled data from 5 experiments display fold change from media-pretreated, media-stimulated; error bars indicate SEM, p-values from one-way ANOVA. Unmarked comparisons were non-significant.

Eosinophils can be stimulated to produce and release IL-1β in a MAPK-dependent manner [55], [56]. IL1β mRNA contains known AU-rich elements (ARE) that are well-defined cis-elements in the 3′ untranslated region of mRNAs that are controlled by ERK1/2 in eosinophils and responsible for mRNA stabilization and accumulation [57], [58]. As cholesterol depletion decreased p-ERK1/2 levels (MAPK signaling, Fig. 4), we tested the hypothesis MβCD would cause a concomitant reduction in *IL-1β* mRNA expression induced by IL-5. PBEos pretreated with MβCD expressed significantly less IL-5-stimulated *IL-1β* mRNA relative to media pretreated, IL-5-stimulated controls (p<0.05, n = 5; Figure 5). Cells treated with MβCD +1%Chol for a no net cholesterol change responded to IL-5 stimulation with increases in *IL-1β* mRNA levels (p<0.01 for IL-5 stimulation) similar to media-treated controls (no difference with p>0.05, n = 5; Figure 5). Pretreatment with MβCD +2%Chol to increase membrane cholesterol similarly did not alter basal levels or IL-5 induced *IL-1β* mRNA compared with control (p<0.05 for IL-5 induction). These data are consistent with the reduction in p-ERK1/2 and p-p38 following cholesterol depletion (4), and a model in which *IL-1β* mRNA production is regulated by MAPK signaling.

### Elevated cellular membrane cholesterol down-regulated IL-5-dependent cyclin-D3 expression

**Figure 6:**
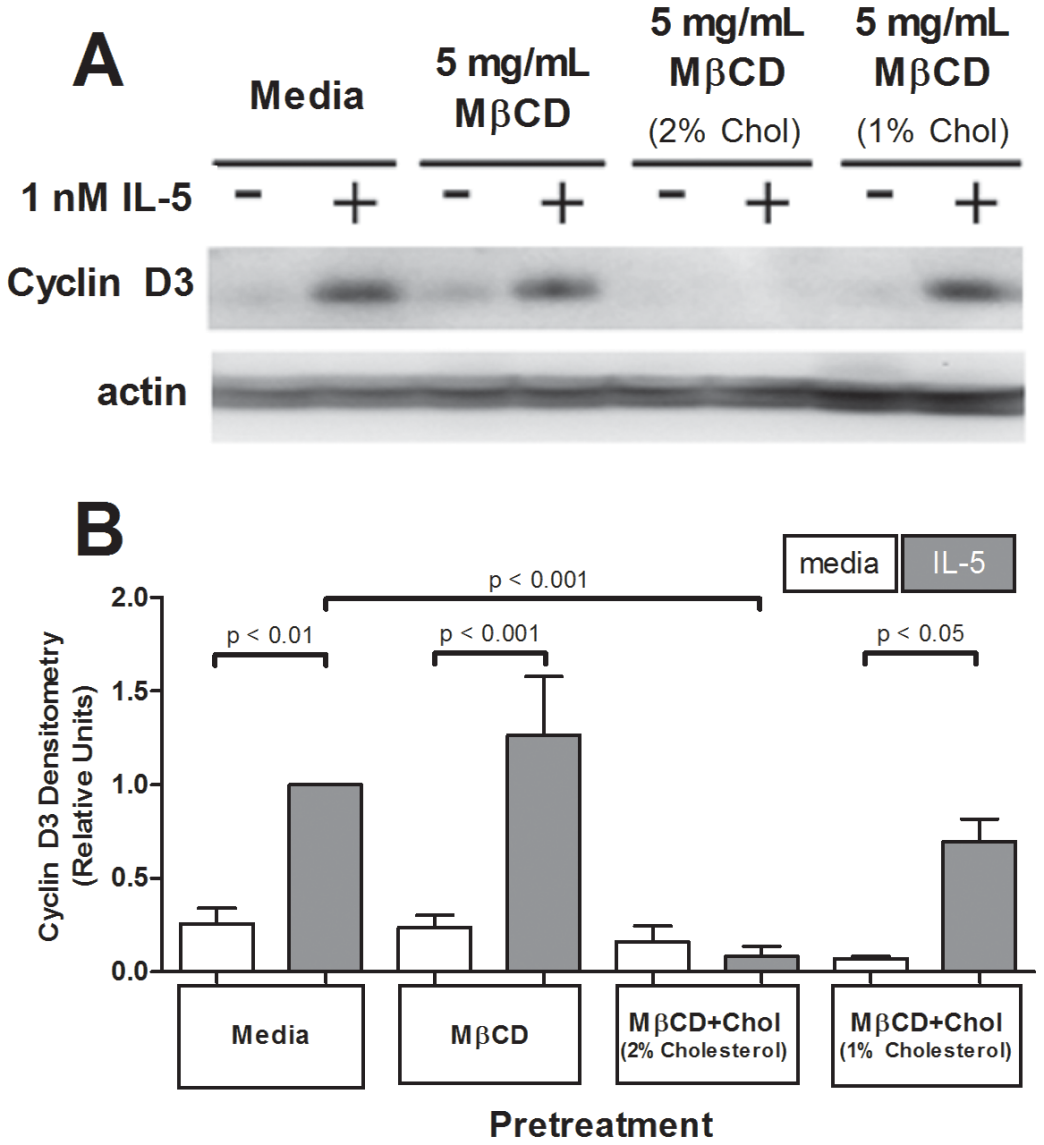
Cholesterol addition reduced IL-5-induced cyclin D3 expression. One million eosinophils per treatment were pretreated 1 hour with media, 5 mg/mL MβCD, 5 mg/mL MβCD+2%Chol, or 5 mg/mL MβCD+1%Chol, washed, then stimulated with media or 1 nM IL-5 for 16 hours. (**A**) Sample lysates were immunoblotted for cyclin D3. (**B**) Pooled data from 5 experiments represented as ratio relative to media pretreated (normalized to actin loading), IL-5-stimulated. Error bars indicate SEM, p-values from one-way ANOVA. Unmarked comparisons were non-significant.

As the human *cyclin D3* gene has upstream consensus sequences for both STAT and MEK/ERK-regulation activating protein 1 promoter regions [59], we tested the hypothesis that cyclin D3 expression would be sensitive to cholesterol manipulation. Cholesterol depletion (MβCD) did not significantly alter IL-5-stimulated increase in cyclin D3 protein expression compared to media-pretreated controls (Figure 6A and B), though protein levels trended upward. The addition of membrane cholesterol via MβCD+2%Chol-pretreatment, however, dramatically reduced IL-5-induced cyclin D3 protein expression to almost undetectable levels (p<0.001, n = 5; Figure 6A and B). MβCD+1%Chol (no net cholesterol change) had no effect on IL-5-induced expression of cyclin D3 compared to media pretreated controls (p>0.05, n = 5), resulting in increased cyclin D3 protein expression following IL-5 stimulation (p<0.05, n = 5; Figure 6A and B) similar to the trend seen in control cells.

### Elevated cellular membrane cholesterol attenuated IL-5-induced metabolic activity and enhanced caspase 3 activation despite IL-5-stimulation

**Figure 7:**
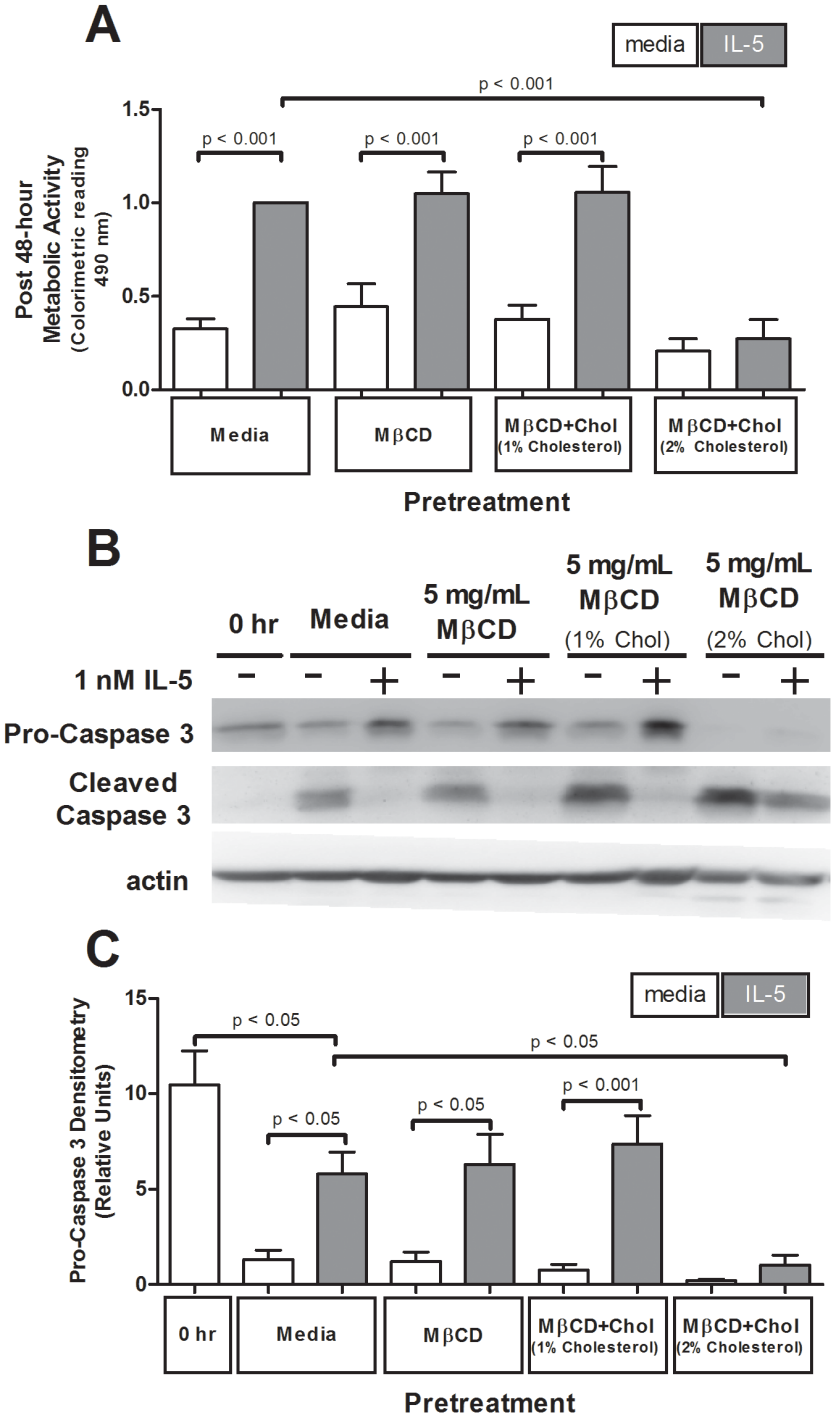
Cholesterol addition reduced IL-5-induced eosinophil survival and enhanced caspase 3 cleavage. One million eosinophils per treatment were pretreated 1 hour with media, 5 mg/mL MβCD, 5 mg/mL MβCD+1%Chol or 5 mg/mL MβCD+2%Chol, washed, then stimulated with media or 100 pM IL-5. (**A**) Viability was assessed via MTS assay after 48 hours. Pooled data from 4 experiments represented as fold change from media pretreated, IL-5-stimulated. (**B**) After 24 hours, pre-treated and stimulated PBEos were lysed and immunoblotted for caspase 3 (pro- and active/cleaved form). (**C**) Pooled data from 5 experiments represented as a ratio of pro-caspase 3 to cleaved caspase 3. Error bars indicate SEM, p-values from one-way ANOVA. Unmarked comparisons were non-significant.

To determine how membrane cholesterol manipulation regulates eosinophil survival, we used MTS assays to quantify metabolic activity as a survival indicator. As shown in Figure 7A, neither cholesterol reduction (MβCD pretreatment) nor no-cholesterol change (MβCD+1%Chol pretreatment) altered IL-5-induced increase in metabolic activity at 48 hours post-treatment (p<0.001 each, n = 4). In contrast, elevated membrane cholesterol (MβCD+2%Chol pretreatment) attenuated IL-5-induced 48-hour survival relative to media pretreatment (p<0.001, n = 4; Figure 7A). Loss of IL-5-induced cellular metabolism, despite consistent STAT phosphorylation (Fig. 3 above), suggests an alternate, unidentified mechanism exists regulating the otherwise classic IL-5-induced eosinophil survival.

To determine whether this loss of survival corresponded with activation of apoptotic pathways, we quantified caspase 3 cleavage (activation) as a ratio of pro- to cleaved-caspase 3 in lysates from PBEos harvested 24 hours post-cholesterol treatment and subsequent IL-5 stimulation. In media pre-treated cells, 24 hour IL-5 stimulation significantly elevated the pro∶cleaved caspase 3 ratio compared non-stimulated-controls (p<0.05, n = 5; Figure 7B and C), indicating decreased caspase 3 cleavage, consistent with an IL-5 induced increase in cell survival. IL-5 stimulation likewise elevated the pro∶cleaved caspase 3 ratio after MβCD pretreatment (p<0.05, n = 5; Figure 7C) or MβCD+1%Chol pretreatment (p<0.001, n = 5; Figure 7C). Pretreatment with MβCD+2%Chol to increase membrane cholesterol, however, resulted in nearly complete cleavage of pro-caspase 3 in both IL-5 stimulated and unstimulated conditions (Figure 7B), which significantly decreased pro∶cleaved ratio relative to media pretreated control cell lysates (p<0.05, n = 5; Figure 7C). These data indicate cholesterol addition stimulated caspase 3 activation, suggesting exogenous cholesterol increases PBEos cell death regardless of the generally pro-survival IL-5 stimulus and retained STAT activation.

## Discussion

To determine whether eosinophil inflammatory responses are sensitive to cholesterol regulation, we defined the effects that altering cell membrane cholesterol content has on specific eosinophil signaling pathways. Depleting cellular cholesterol *in vitro* attenuated IL-5-induced p38 and MEK/ERK phosphorylation, and *IL-1β* mRNA increases in human eosinophils without altering surface expression levels of the IL-5 receptor. Exogenous cholesterol supplementation elevated basal p38 activation, and attenuated IL-5-induced increases in cyclin D3 protein expression and total cellular metabolic activity. Neither manipulation altered IL-5-induced JAK/STAT signaling, as assayed by STAT3 and STAT5 phosphorylation, importantly demonstrating there was not a global downregulation of eosinophil signaling. These data suggest membrane cholesterol composition selectively regulates IL-5-induced signaling events that are dependent upon membrane-anchored signaling proteins, with further specificity highlighted by the differential responses between MEK/ERK and p38. Future studies will identify the proteins that confer cholesterol sensitivity to the MAPK pathways, with likely candidates including membrane-anchored Raf and Lyn, which act upstream of p38 and ERK1/2.

Selective, cholesterol-dependent sensitivity of the MEK/ERK pathway in response to IL-5 contrasts cholesterol-independent JAK/STAT signaling, and is consistent with the study by Lei et al demonstrating the localization of IL-5Rs to membrane microdomains defines which intracellular signaling proteins are bound to the receptor [43]. Completely unexpected, however, were our findings that the addition of exogenous cholesterol dramatically increased basal p38 phosphorylation to levels equivalent to IL-5 stimulation. These data suggest that eosinophils exhibiting elevated membrane cholesterol as part of a disease state will functionally express constitutively active p38. In addition, such eosinophils are predicted to manifest decreased responsiveness to IL-5 as the pre-phosphorylated p38 precluded further IL-5 activation.

IL-5 stimulation triggers events that promote long-term survival of eosinophils, which otherwise undergo significant levels of apoptosis within 48 hours of *in vitro* culture [60]. Survival contributes to overall eosinophil inflammatory capacity. Our data demonstrate, however, that cholesterol supplementation increased caspase 3 cleavage *despite* IL-5 stimulation, suggesting an additional, unidentified protein(s) or pathway(s) regulates eosinophil apoptotic signaling in a cholesterol-dependent manner. It is possible that cholesterol (non-cytokine)-induced phospho-p38 (active p38) is responsible for attenuating IL-5-mediated eosinophil survival, as previous studies demonstrate that p38 promotes caspase 3 cleavage under specific conditions, leading to apoptosis [61]. Our data predict a model in which p38 activated by a non-cytokine source triggers apoptosis, regardless of IL-5 stimulation and persistent JAK/STAT survival signals. This model further predicts that inhibiting basal p38 phosphorylation in the presence of exogenous cholesterol would restore IL-5-induced cell survival.

Phosphorylated p38 may also be responsible for the decrease in cyclin D3 protein levels observed in cells treated with exogenous cholesterol. While the functional role of cyclin D3 in terminally differentiated and tissue human eosinophils is unclear, recent studies demonstrate function likely extends beyond cell cycle control [19], [28]. Future experiments may determine whether cholesterol-induced phospho-p38 promotes cyclin D3 protein degradation or prevents IL-5 mediated increased production, and may provide a mechanism for fine-tuning IL-5-induced cyclin D3 protein expression.

Additional studies will also be required to determine the mechanism by which exogenous cholesterol increased basal p38 phosphorylation. One potential mechanism may be cytokine-independent interactions between p38 and the scaffold protein TAB1, which can activate p38 in PBEos [62]. The cellular consequences of increasing baseline levels of p-p38 in huPBEos similarly remains to be determined, but a genome study in mouse embryonic kidney cells found that p38 activation up-regulates a distinct array of genes such that each subset of downstream effectors is specific to the upstream p38-activation source (*i.e.*, osmostress, cytokine, or protein synthesis) [63]. The genomic study suggests cholesterol-based p38 activation regulates an array of genes distinct from IL-5 induced p38 activation.

IL-1β is a key inflammatory cytokine, traditionally viewed as a product of monocytes and macrophages, but recent studies have demonstrated human eosinophils also have the capacity to release active IL-1β that increases IL-17 expression by CD4+ T lymphocytes [45]. IL-1-β production and release are controlled at many different levels. IL-1β mRNA accumulation depends on its stability and the MAP kinase activity [64]. Our data support that reduced MAP kinase activation due to depletion of cholesterol subsequently decreases the level of IL-1β mRNA. Synthesis and extracellular release of mature IL-1β requires two coordinate signals in monocytes and macrophages. The second signal to complement IL-5 for the production and the release of a mature IL-1β protein from eosinophils remains unknown. Controlled cholesterol depletion may provide an excellent tool for dissecting the mechanism of IL-1β expression and secretion in eosinophils, without altering other key cellular aspects, including viability.

One limitation of the current approach is that while MβCD is considered a cholesterol chelator, particularly given the dose and incubation times used in the study, MβCD treatment may deplete cholesterol from the entire cell membrane rather than solely cholesterol-rich lipid rafts. At high doses (typically >10 mM), MβCD may also extract phospholipids and membrane proteins [65]. In a complimentary approach, we treated cells overnight with eicosapentaenoic acid (EPA), a polyunsaturated fatty acid that alters lipid raft structure and composition without depleting total cellular cholesterol. Treating cells with EPA therefore distinguishes whether MβCD -induced changes in cell signaling are due to alterations in total membrane cholesterol or membrane organization. EPA treatment resulted in increased basal p38 phosphorylation and a concomitant loss of IL-5 responsiveness in eosinophils, mimicking the effects of increasing cholesterol with MβCD +2%Chol. Whether EPA treatment redistributes cholesterol across the membrane, eliminating lipid rafts, or in fact forms larger, more ordered lipid rafts appears to be condition- and cell type-dependent [51]–[54], [66]. Since cholesterol addition enhances the formation of cholesterol-rich lipid rafts [67], [68], these data suggest that 33 µM, EPA likely increases the size and organization of lipid rafts in human eosinophils, a hypothesis that can be tested in future studies. The data furthermore demonstrate that the changes in p38 phosphorylation induced by cholesterol manipulation are due to altered membrane structure, rather than being dependent solely upon total cholesterol concentration. Future studies may refine the specific role of membrane architecture in eosinophil signaling, and potentially distinguish various classes of cell membrane microdomains including sphingolipid-rich, caveolae, tetraspanin-rich, and galectin lattices [69]–[72].

The cholesterol-mediated regulation of eosinophil inflammatory capacity may reflect physiological conditions in which there is (1) enhanced HDL-packaging and cholesterol export, (2) hypercholesterolemia, or (3) hypercholesterolemia treated utilizing fish oil or cholesterol-lowering drugs like stating. Statins can reduce high serum cholesterol levels (clinically defined as >240 mg/dl) to levels as low as 70 mg/dl, and circulating eosinophils are directly exposed to those serum cholesterol concentrations. Though there is not currently a defined stoichiometry between circulating and cell membrane cholesterol levels, tumor cells isolated from mice fed a high-cholesterol diet for 4 weeks had a cholesterol/protein ratio (ug/mg) twice that of cells from animals fed a normal diet [73]. In the same study, cultured cells treated with 10 µM Simvastatin had a 3× reduction in the cholesterol/protein ratio. The 25% reduction in eosinophil membrane cholesterol achieved with 5 mg/mL MβCD, and the 2× increase with our 2% exogenous cholesterol treatment therefore likely fall within a clinically-relevant physiological range. It is reasonable to predict that eosinophils isolated from human subjects over a range of normal and relevant cholesterol disease states would exhibit concomitant signaling changes that correlate with circulating cholesterol levels and parallel the data presented here.

Our data demonstrate human eosinophil signaling pathways are selectively sensitive to the levels of cellular membrane cholesterol, likely altering membrane architecture. Numerous diseases linked to loss of optimal cholesterol homeostasis involve eosinophils as part of the inflammatory response and disease progression, including atherosclerosis and Alzheimer's [1]–[10], and recent studies have assessed the relationship between hypercholesterolemia and low levels of serum high density lipoprotein (HDL) and risk for asthma [35], [37], [40], [41]. Future studies will define how the eosinophil responses to cholesterol levels relate to human disease states.

## Supporting Information

Figure S1 **Cholesterol manipulation did not alter eosinophil size or density as determined via flow cytometry.** Scatter plots of side scatter versus forward scatter of eosinophils pretreated 1 hour with *i*. media, *ii*. 5 mg/mL MβCD, or *iii*. 5 mg/mL MβCD+2%Chol (3–5×10^5^ per treatment).
(TIF)

Figure S2 **Pretreatment with reduced percentage of cholesterol in MβCD+Chol (1% cholesterol) resulted in no net-change in PBEos cellular membrane cholesterol.** (**A**–**B**) Eosinophils were treated 1 hour with media, 5 mg/mL MβCD, 5 mg/mL MβCD+2%Chol, or 5 mg/mL MβCD+1%Chol (3–5×10^5^ per treatment). Cells were fixed, stained with 50 µg/mL Filipin III for cholesterol, then analyzed via flow cytometry. (**A**) Representative histogram from n = 5 experiments of cells stained with FIII after treatment with *i*. Media, *ii*. MβCD, *iii*. MβCD+2%Chol, or *iv*. MβCD+1%Chol. (**B**) Quantification of 5 independent experiments of FIII-stained eosinophils after treatment with concentrations indicated above. Error bars indicate SEM, p-values from one-way ANOVA. (**C**–**D**) One million eosinophils per treatment were pretreated 1 hour with media, 5 mg/mL MβCD +2%Chol, or 5 mg/mL MβCD +1%Chol, then stimulated +/− IL-5 for 15 min. (**C**) Samples were immunoblotted for active p38. (**D**) Pooled data (n = 5) normalized to actin loading, error bars indicate SEM, p-values from non-parametric one-way ANOVA. Irrelevant lanes were digitally removed from between lanes 2 and 3. Unmarked comparisons were non-significant.
(TIF)

Figure S3 **EPA treatment increases basal p38 phosphorylation, eliminating an IL-5-stimulated increase.** One million eosinophils per treatment pretreated 18 hours with media or 33 uM EPA were stimulated +/− IL-5 for 15 min. (**A**) Western blots were probed for phosphorylated STAT5 or p38, and reprobed with actin antibodies as a loading control. Example blot is representative of n = 3 experiments. (**B**) Graph plots p-p38 band density normalized to actin as mean +/− SE for each treatment. Data are pooled from 3 experiments, and ctl + IL-5 samples were set as 1. (**C**) Graph plots p-STAT5 band density normalized to actin as mean +/− SE for each treatment. Data are pooled from 3 experiments, and only IL-5-stimulated bands were quantified as p-STAT5 was undetectable in lysates from unstimulated cells.
(TIF)
