# Supplementary figures and images for: Cholesterol Selectively Regulates IL-5 Induced Mitogen Activated Protein Kinase Signaling in Human Eosinophils
Source: PLoS One. 2014 Aug 14;9(8):e103122. doi: 10.1371/journal.pone.0103122 (PMC4133209; doi:10.1371/journal.pone.0103122)

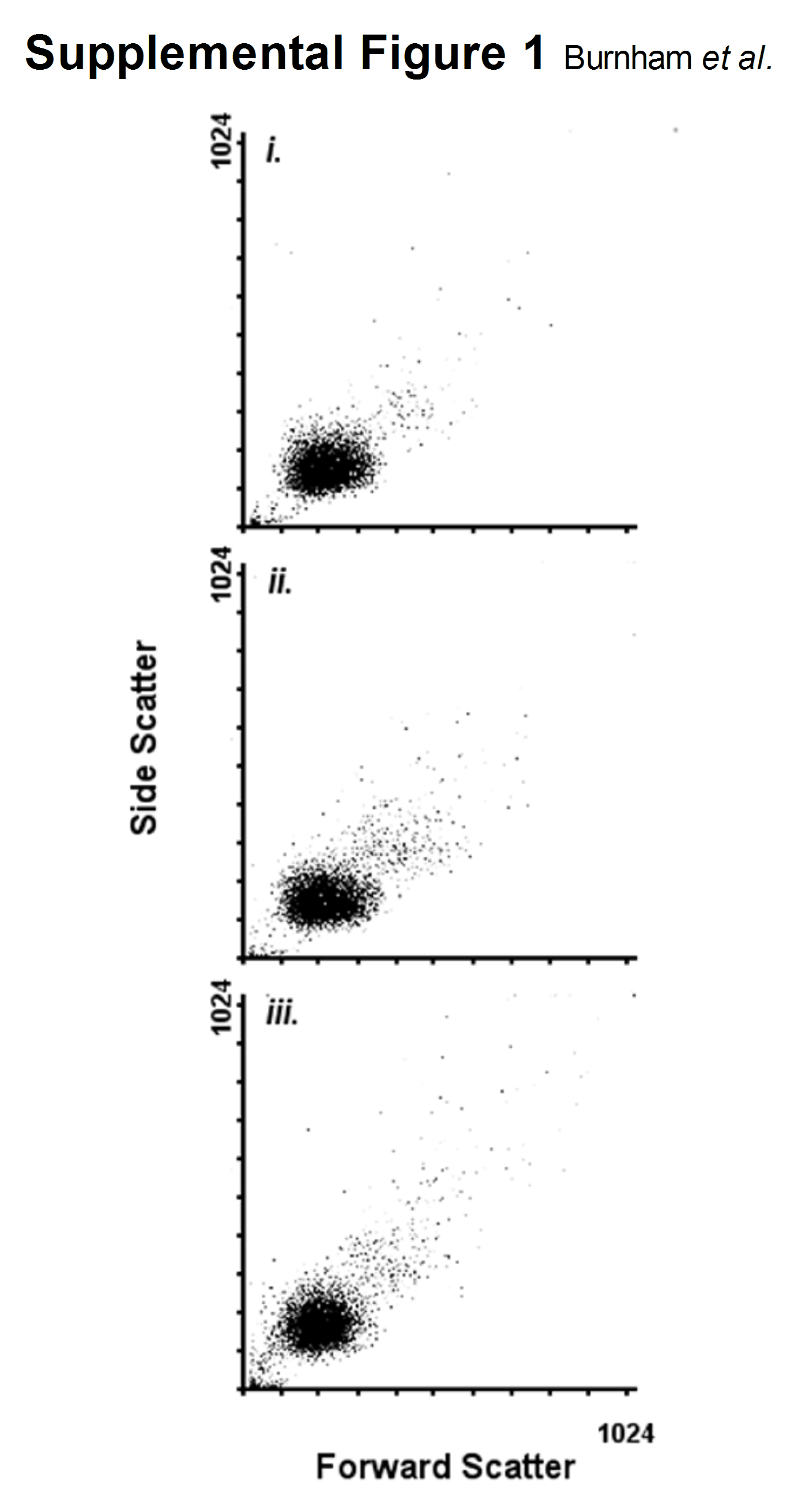

Supplement: Figure S1 — Cholesterol manipulation did not alter eosinophil size or density as determined via flow cytometry. Scatter plots of side scatter versus forward scatter of eosinophils pretreated 1 hour with i. media, ii. 5 mg/mL MβCD, or iii. 5 mg/mL MβCD+2%Chol (3–5×10^5^ per treatment). (TIF) [file pone.0103122.s001.tif]

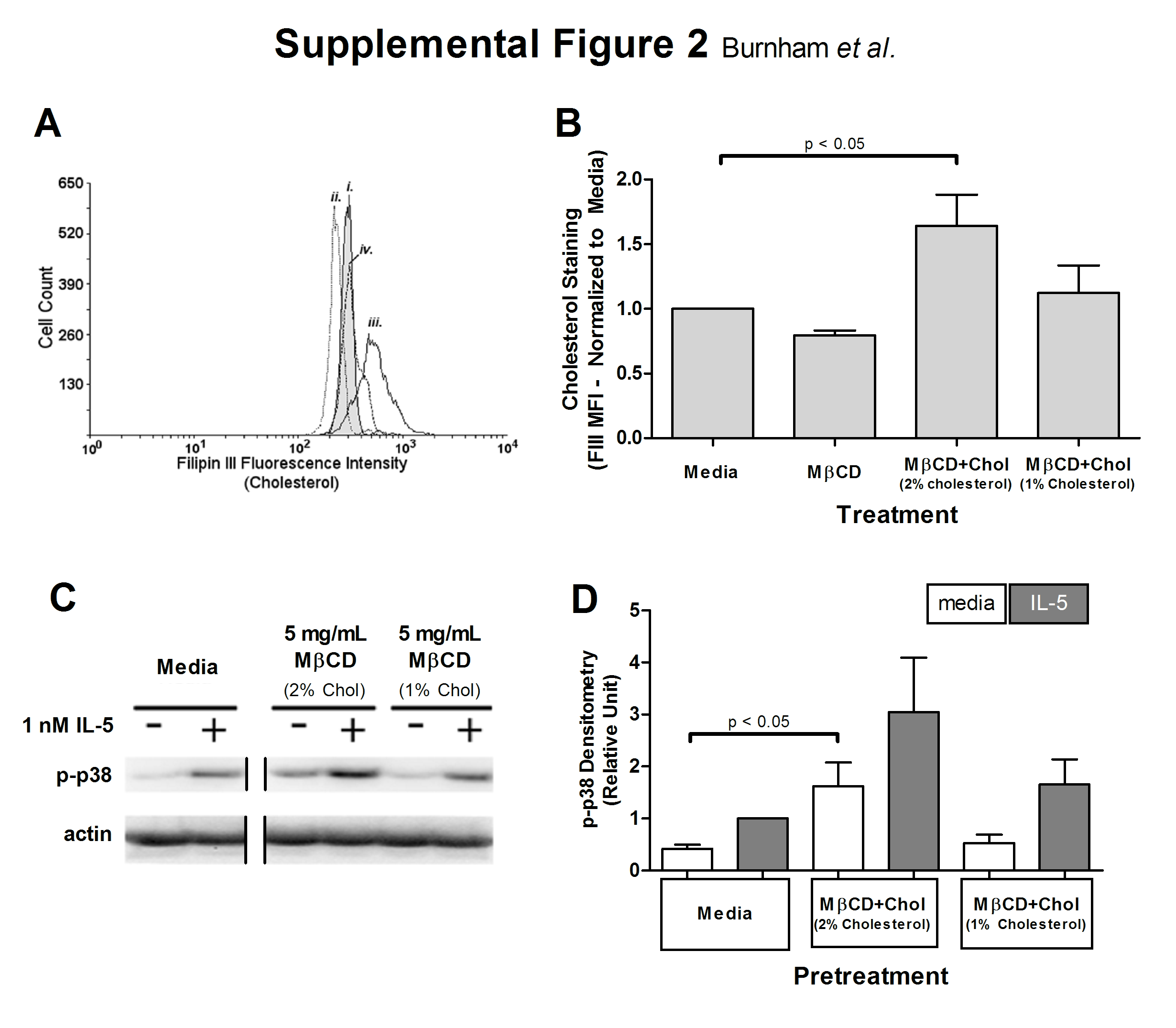

Supplement: Figure S2 — Pretreatment with reduced percentage of cholesterol in MβCD+Chol (1% cholesterol) resulted in no net-change in PBEos cellular membrane cholesterol. (A–B) Eosinophils were treated 1 hour with media, 5 mg/mL MβCD, 5 mg/mL MβCD+2%Chol, or 5 mg/mL MβCD+1%Chol (3–5×10^5^ per treatment). Cells were fixed, stained with 50 µg/mL Filipin III for cholesterol, then analyzed via flow cytometry. (A) Representative histogram from n = 5 experiments of cells stained with FIII after treatment with i. Media, ii. MβCD, iii. MβCD+2%Chol, or iv. MβCD+1%Chol. (B) Quantification of 5 independent experiments of FIII-stained eosinophils after treatment with concentrations indicated above. Error bars indicate SEM, p-values from one-way ANOVA. (C–D) One million eosinophils per treatment were pretreated 1 hour with media, 5 mg/mL MβCD +2%Chol, or 5 mg/mL MβCD +1%Chol, then stimulated +/− IL-5 for 15 min. (C) Samples were immunoblotted for active p38. (D) Pooled data (n = 5) normalized to actin loading, error bars indicate SEM, p-values from non-parametric one-way ANOVA. Irrelevant lanes were digitally removed from between lanes 2 and 3. Unmarked comparisons were non-significant. (TIF) [file pone.0103122.s002.tif]

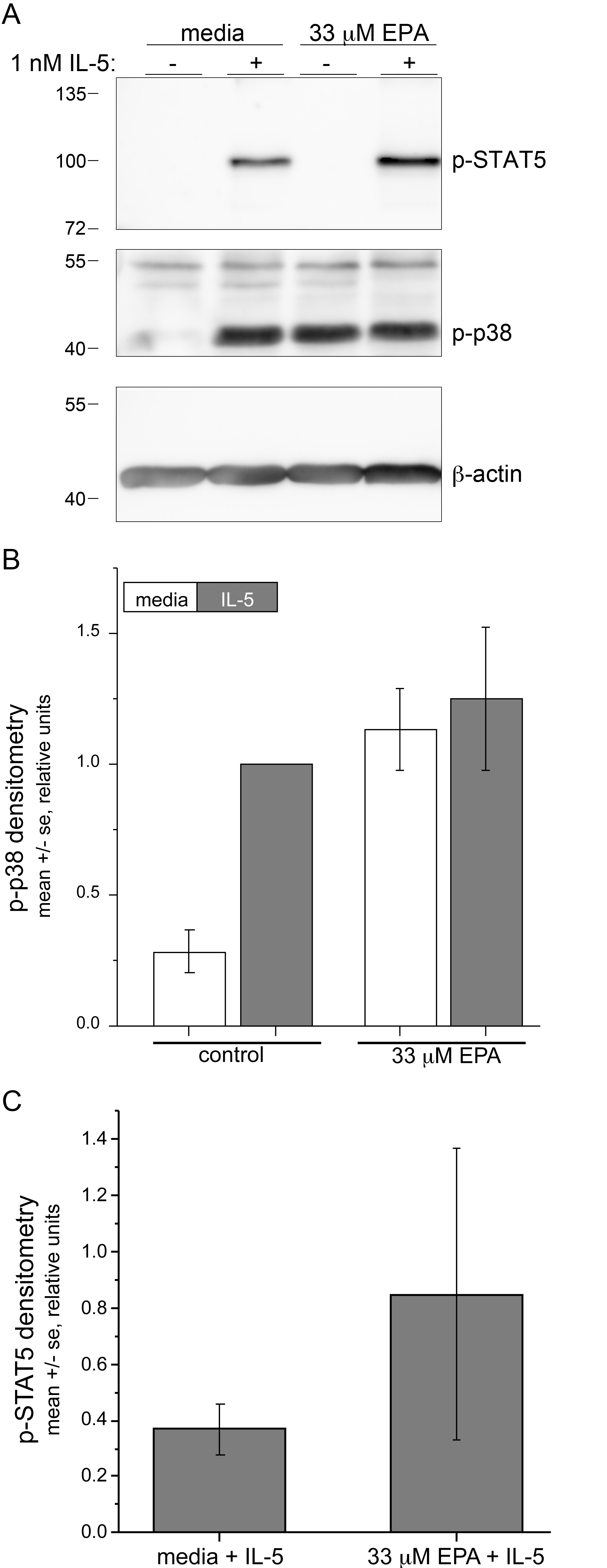

Supplement: Figure S3 — EPA treatment increases basal p38 phosphorylation, eliminating an IL-5-stimulated increase. One million eosinophils per treatment pretreated 18 hours with media or 33 uM EPA were stimulated +/− IL-5 for 15 min. (A) Western blots were probed for phosphorylated STAT5 or p38, and reprobed with actin antibodies as a loading control. Example blot is representative of n = 3 experiments. (B) Graph plots p-p38 band density normalized to actin as mean +/− SE for each treatment. Data are pooled from 3 experiments, and ctl + IL-5 samples were set as 1. (C) Graph plots p-STAT5 band density normalized to actin as mean +/− SE for each treatment. Data are pooled from 3 experiments, and only IL-5-stimulated bands were quantified as p-STAT5 was undetectable in lysates from unstimulated cells. (TIF) [file pone.0103122.s003.tif]
